# Closely related phytoplankton species produce similar suites of dissolved organic matter

**DOI:** 10.3389/fmicb.2014.00111

**Published:** 2014-03-28

**Authors:** Jamie W. Becker, Paul M. Berube, Christopher L. Follett, John B. Waterbury, Sallie W. Chisholm, Edward F. DeLong, Daniel J. Repeta

**Affiliations:** ^1^Department of Biology, Woods Hole Oceanographic InstitutionWoods Hole, MA, USA; ^2^Department of Civil and Environmental Engineering, Massachusetts Institute of TechnologyCambridge, MA, USA; ^3^Department of Marine Chemistry and Geochemistry, Woods Hole Oceanographic InstitutionWoods Hole, MA, USA; ^4^Department of Biology, Massachusetts Institute of TechnologyCambridge, MA, USA; ^5^Department of Biological Engineering, Massachusetts Institute of TechnologyCambridge, MA, USA

**Keywords:** dissolved organic matter, untargeted metabolomics, marine phytoplankton, exometabolome, *Prochlorococcus*, *Synechococcus*, *Thalassiosira*, *Phaeodactylum*

## Abstract

Production of dissolved organic matter (DOM) by marine phytoplankton supplies the majority of organic substrate consumed by heterotrophic bacterioplankton in the sea. This production and subsequent consumption converts a vast quantity of carbon, nitrogen, and phosphorus between organic and inorganic forms, directly impacting global cycles of these biologically important elements. Details regarding the chemical composition of DOM produced by marine phytoplankton are sparse, and while often assumed, it is not currently known if phylogenetically distinct groups of marine phytoplankton release characteristic suites of DOM. To investigate the relationship between specific phytoplankton groups and the DOM they release, hydrophobic phytoplankton-derived dissolved organic matter (DOM_P_) from eight axenic strains was analyzed using high-performance liquid chromatography coupled to mass spectrometry (HPLC-MS). Identification of DOM features derived from *Prochlorococcus*, *Synechococcus*, *Thalassiosira*, and *Phaeodactylum* revealed DOM_P_ to be complex and highly strain dependent. Connections between DOM_P_ features and the phylogenetic relatedness of these strains were identified on multiple levels of phylogenetic distance, suggesting that marine phytoplankton produce DOM that in part reflects its phylogenetic origin. Chemical information regarding the size and polarity ranges of features from defined biological sources was also obtained. Our findings reveal DOM_P_ composition to be partially conserved among related phytoplankton species, and implicate marine DOM as a potential factor influencing microbial diversity in the sea by acting as a link between autotrophic and heterotrophic microbial community structures.

## Introduction

Extracellular release of dissolved organic matter (DOM) by marine phytoplankton fuels secondary production in the sea (Pomeroy, 1974; Mague et al., 1980; Fogg, 1983; Baines and Pace, 1991). As much as 50% of the total carbon fixed by photoautotrophs may be released into seawater as a by-product of normal metabolism, or through active processes for waste removal, substrate acquisition, defense, or communication (Bjornsen, 1988; Carlson, 2002; Bertilsson and Jones, 2003). The amount of extracellular DOM released and its composition depend on the organism and its physiological state (Myklestad, 1995; Meon and Kirchman, 2001; Wetz and Wheeler, 2007; Romera-Castillo et al., 2010), as well as additional factors including: temperature, light, growth phase, availability of inorganic nutrients, and the presence of other organisms (Hirt et al., 1971; Obernosterer and Herndl, 1995; Grossart and Simon, 2007; Barofsky et al., 2009; Engel et al., 2011). Factors affecting DOM uptake are less clear, but recent evidence suggests that DOM resource partitioning within microbial communities may impact the ecological success and community structure of heterotrophic bacterioplankton (McCarren et al., 2010; Poretsky et al., 2010; Nelson and Carlson, 2012; Sarmento and Gasol, 2012). Substrate specificity may be of particular importance for heterotrophs that have evolved streamlined genomes and are therefore fine-tuned to particular resources (Giovannoni et al., 2005, 2008).

Genomic and physiological differences among marine phytoplankton likely influence the nature of the organic matter they produce, and consequently, what substrates are available to sympatric heterotrophic communities. If different phytoplankton groups release DOM of varying composition and lability, then DOM could provide a direct link between autotrophic and heterotrophic microbial diversity and community structure. For example, *Prochlorococcus* is frequently the numerically dominant photoautotroph in oligotrophic surface waters and is an essential source of organic matter used by heterotrophic bacterioplankton in this environment (Partensky et al., 1999; Bertilsson et al., 2005; Flombaum et al., 2013). It has been suggested that DOM released by *Prochlorococcus* could be responsible for supporting 12–41% of bacterial production in tropical and subtropical regions (Bertilsson et al., 2005). If DOM released by *Prochlorococcus* is distinct in composition, metabolic specialization to utilize *Prochlorococcus*-derived DOM within associated heterotrophic populations may influence bacterioplankton community structure in these regions.

Several recent studies have employed phytoplankton-derived DOM for use in the study of microbial DOM consumption (Romera-Castillo et al., 2011; Nelson and Carlson, 2012; Sarmento and Gasol, 2012; Landa et al., 2013a,b; Sharma et al., 2013), with the implied assumption that DOM suites derived from individual phytoplankton strains represent compositionally distinct organic substrates. However, a comparative assessment of phytoplankton-derived DOM has not been conducted at the molecular level and it is not yet clear how much chemical variation exists in DOM produced by different marine phytoplankton types. Furthermore, it is not known how DOM composition varies as a function of taxonomic or phylogenetic relatedness among different phytoplankton species. Investigating how DOM produced by marine phytoplankton varies with phylogenetic relatedness may help us to better understand marine microbial community structure, allow us to target important components of DOM in future studies, and potentially improve our predictive powers regarding the composition of DOM produced by different phytoplankton communities.

To investigate the relationship between phylogenetic relatedness and DOM composition, we used HPLC-MS to detect chemical features in DOM released by laboratory cultures of marine phytoplankton. While the determination of empirical formulae within DOM is now possible through advances in ultrahigh-resolution mass spectrometry, definitive chemical identifications in untargeted metabolomic footprinting studies remain a challenge due to structural complexities including isomerization and the magnitude of unknown metabolites that comprise both marine (Nebbioso and Piccolo, 2013) and phytoplankton-derived DOM (Schwarz et al., 2013). Rather than attempt chemical identifications with no a priori knowledge regarding compounds of interest, we chose to sacrifice mass resolution for chromatographic separation, providing us with two independent parameters (i.e., polarity and mass) to detect and compare chemical features in DOM released by eight marine phytoplankton strains. These strains were selected to encompass both the Bacteria and Eukarya domains. Cyanobacterial strains within the genera *Prochlorococcus* and *Synechococcus* were chosen to represent cells with different adaptations to light and temperature. *Thalassiosira* and *Phaeodactylum* were chosen to represent different groups of eukaryotic diatoms (centric and pennate, respectively). Selection of these strains provided both broad phylogenetic coverage as well as examples of closely related organisms with unique physiologies. Hundreds to thousands of features detected in each culture sample were used to chemically compare DOM produced by phytoplankton strains with differing degrees of relatedness. The suite of features associated with each strain was then compared with phylogenies deduced from both rRNA and whole genome comparisons to uncover novel connections between phytoplankton phylogeny and DOM composition.

## Materials and methods

### Phytoplankton cultures

Eight species of marine phytoplankton were selected for this study to investigate variability in the composition of organic matter released by unicellular photoautotrophs at various taxonomic levels (Table 1). Individual strains were chosen on the criteria that a published sequenced genome is, or will soon be available, and that axenic strains were capable of growth to high cell densities (ca. 10^7^ cells/ml) in natural seawater-based media. All media was prepared by the National Center for Marine Algae and Microbiota (NCMA) using filtered, autoclaved surface water collected from the Sargasso Sea. Media amendments were added aseptically after sterilization. Triplicate 1 L batch cultures of each strain were prepared alongside triplicate 1 L controls containing all media amendments, but no cell additions. Cells were pre-conditioned in 60–400 ml of the appropriate medium prior to inoculation in 1 L.

**Table 1:** **DOM_P_ features detected using HPLC-ESI-MS after blank subtraction**.

**Phytoplankton**	**Features**			**Unique features**			**Common features**	**Total features**
	**Replicate A**	**Replicate B**	**Replicate C**	**Replicate A**	**Replicate B**	**Replicate C**		
*Prochlorococcus*^a^							43	328
MIT9313	321	303	307	97	128	121	123	577
MIT9301	449	430	459	72	130	38	232	673
MED4	NA	346	247	NA	216	117	130	463
*Synechococcus*^b^							68	483
WH8102	642	586	516	76	57	58	389	773
WH7803	299	324	255	47	44	61	162	434
Diatoms^c^							31	1478
CCMP632	876	918	1026	60	42	151	693	1190
CCMP1647	696	956	543	44	288	38	435	1065
CCMP1335	832	757	659	139	56	44	541	973

*Feature totals for individual biological replicates of each phytoplankton strain are shown along with the sum of all features for each strain. The number of features unique to an individual replicate as well the number of features common to all replicates for a given strain are also included. Total and common features (given that each feature was shared among biological replicates) are provided for each organism group*.

a *Described in Moore and Chisholm (1995); Moore et al. (1998); Rubio et al. (1999); Rocap et al. (2002, 2003)*.

b *Described in Waterbury et al. (1986); Rocap et al. (2002); Palenik et al. (2003)*.

c *Described in Sorhannus (2004); De Martino et al. (2007); Alverson et al. (2011)*.

*Thalassiosira pseudonana* (CCMP str. 1335), *Thalassiosira rotula* (CCMP str. 1647), *Phaeodactylum tricornutum* (CCMP str. 632), and both *Synechococcus* strains (WH8102 and WH7803) were grown at the NCMA in L1 medium prepared according to existing protocols (Guillard and Hargraves, 1993). Diatom strains were grown at 20°C and *Synechococcus* strains at 24°C. All five strains were grown on a 13/11 light/dark cycle with gentle periodic mixing. *Synechococcus* str. WH7803 was grown under 10–20 μmol photons/m^2^/s illumination, while the remaining strains were grown at 100–120 μmol photons/m^2^/s. Diatom cells were enumerated using a Palmer-Maloney counting chamber at 40X magnification on a Zeiss light microscope. *Synechococcus* cells were enumerated via epifluorescence microscopy. Three *Prochlorococcus* strains (MED4, MIT9301, and MIT9313) were grown at 22°C in Pro99 medium prepared by the NCMA according to existing protocols (Moore et al., 2002, 2007) with the addition of 5 mM sodium bicarbonate. All three strains were grown on a 14/10 light/dark cycle. *Prochlorococcus* str. MIT9313 was grown under low light conditions at ca. 20 μmol photons/m^2^/s, while MIT9301 and MED4 were grown at ca. 40 μmol photons/m^2^/s. *Prochlorococcus* cultures were monitored for growth using total fluorescence and for changes in pH, which were mitigated by additions of 2 mM HEPES buffer and 5 mM sodium bicarbonate 6 days after inoculation (sensu Moore et al., 2007). Samples for direct cell counts by flow cytometry were fixed with 0.125% final concentration of grade I glutaraldehyde (Sigma) and stored at –80°C prior to analysis. Fixed samples were diluted in 0.2 μm filtered seawater and *Prochlorococcus* were enumerated using an Influx Cell Sorter (BD Biosciences) as previously described (Olson et al., 1985; Cavender-Bares et al., 1999).

### Organic matter production

Samples for cell enumeration and particulate and dissolved organic carbon (POC and DOC, respectively) quantification were taken at the onset of stationary phase growth to evaluate organic carbon production by each strain. An exception was that DOC data for *Prochlorococcus* strains was not acquired due to the addition of HEPES buffer during growth to mitigate pH changes (sensu Moore et al., 2007). All organic carbon samples were processed using combusted (450°C for 8 h) glassware. Duplicate samples for POC analysis were taken by vacuum filtering 25 ml of sample onto a combusted 25 mm 0.7 μm glass fiber filter (Whatman GF/F). Filters were then placed inside a combusted glass petri dish, wrapped in foil, and immediately frozen. A blank filter was also prepared at each sampling point. Filters were later thawed and dried (60°C; overnight) before encapsulation into 9 × 10 mm tin capsules. Measurements for POC quantification were performed by the University of California Davis Stable Isotope Facility. POC filtrates were transferred into combusted glass vials, acidified with 150 μl of a 25% phosphoric acid solution and measured for DOC using high temperature catalytic oxidation on a Shimadzu TOC-V_CSH_ with platinized aluminum catalyst. Sample concentrations were determined alongside potassium hydrogen phthalate standards and consensus reference materials provided by the DOC-CRM program (www.rsmas.miami.edu/groups/biogeochem/CRM.html).

### Extraction of DOM_P_

Upon entering stationary phase, cells were removed by centrifugation at either 878 RCF (diatom cultures) or 10,751 RCF (cyanobacteria cultures) for 15 min, followed by gentle filtration (0.1 μm; Whatman Polycap 36 TC capsule filter). Filtrates were then stored briefly in the dark at 4°C until solid-phase extraction (SPE). Material obtained via SPE constitutes 20–60% of the total DOC in marine DOM (Dittmar et al., 2008), encompassing a large amount of organic substrate available to heterotrophic bacterioplankton. Media controls were processed and stored alongside the culture samples. Filtrates were acidified to pH 2–3 by addition of trace metal grade hydrochloric acid and organic matter was extracted onto ISOLUTE C18(EC) SPE columns (0.5 g, Biotage) at a rate of 1 ml/min. SPE columns were preconditioned with 5 ml HPLC-grade methanol followed by 10 ml ultrapure water. After sampling, mineral salts were washed from the columns with acidified ultrapure water (pH 2–3) at a flow rate of 1 ml/min. Organic matter was recovered by gravity elution using 10 column volumes of acidified HPLC-grade methanol (pH 2–3). Samples were concentrated to a small volume by rotary evaporation, and then taken to dryness under filtered, high purity nitrogen. Samples were resuspended in 156 μl of methanol, and stored briefly in combusted amber vials at 4°C in the dark prior to chemical analysis. A 3 μl subsample of each was placed onto a combusted 25 mm, 0.7 μm glass fiber filter (Whatman GF/F), and submitted for POC analysis to quantify the organic carbon recovered by SPE.

### Chromatography and mass spectrometry

Chromatographic and spectrometric analyses were performed using an Agilent 1200 series high-performance liquid chromatograph coupled to an Agilent 6130 (single quadrupole) mass spectrometer. Organic extracts were separated on a ZORBAX SB-C18 column (Agilent; 3.5 μm 4.6 × 150 mm) eluted at 0.8 ml min^−1^ using a linear gradient (% solvent A, % solvent B, minutes): 100, 0, 0; 20, 80, 31.25; 0, 100, 43.75; 0, 100, 64, where solvent A is aqueous formic acid (0.1%) and solvent B is methanolic formic acid (0.1%). Mass spectrometry was performed using an atmospheric electrospray ionization (ESI) source. Drying gas was set at 11.5 l min^−1^ and 300°C, the nebulizer was at 60 psig, and capillary voltage was set to 4000 V. Data was obtained in the positive ion mode from 100–2000 *m/z* with a 4.0 fragmentor, 150 threshold, and 0.1 step size. A tune solution containing five standard compounds (117–2122 *m/z*) was used to determine mass accuracy of the instrument prior to analysis. Mass accuracy in this size range was found to always be within a tolerance of 0.2 Da.

Mass spectral data was analyzed using MZmine 2 molecular profiling software (Pluskal et al., 2010). Ions with signal intensity at least 5-fold greater than the maximum noise level were identified using a centroid mass detector. Chromatograms were then built from the raw data using a retention time tolerance of ±5 s, and a mass tolerance of ±0.3 *m/z*. Alignments were made to correct for small shifts in retention times between samples and individual peaks were identified by setting a minimum acceptable intensity (5-fold greater than the noise level) and duration (5 s) to remove noise within each chromatogram, and also by searching for local minima within each chromatogram. Adduct peaks ([M+Na]^+^, [M+2Na-H]^+^, [M+H_2_PO_4_]^+^, [M+HSO_4_]^+^) and isotopic peaks were identified and removed within a retention time tolerance of ±1.42 s, and a mass tolerance of ±0.2 *m/z*. Additional adduct peaks for *Prochlorococcus* ([M+NH_4_]^+^) and both *Synechococcus* and diatoms ([M+K]^+^) were identified and removed due to the presence of these chemical species in the media types used to grow these strains. Constraints were placed on the intensity ratio of identified isotope pairs based on the minimum ((M–1)/30) and maximum ((M–3)/14) number of carbon atoms for a given mass. Sample feature lists were then aligned and gaps were filled in using a secondary threshold of 3-fold greater than the noise to identify common peaks that fell just below the initial strict threshold. A feature was defined as a unique *m/z* at a specific retention time, thus multiple features could share a specific *m/z* or retention time, but never both. While the possibility of distinct metabolites sharing identical retention times and *m/z* values within the tolerance levels mentioned above cannot be ruled out, the use of two independent parameters (polarity and mass) to designate DOM_P_ features reduces the likelihood of falsely identifying common features.

Biological triplicates of each strain were first compared against triplicate media controls to distinguish material produced by the organism from any background material, including DOM present in the seawater medium used to cultivate each strain. Features were considered to be associated with a particular organism only if they were present in all three biological replicates of that strain and absent in all three replicate controls of the appropriate media type. Removal of features present in sterile media controls reduced the possibility of differences arising due to variations in growth media (i.e., Pro99 medium vs. L1 medium). Features were also removed if they were present in any blank samples, including triplicate instrument blank injections of pure methanol and triplicate processing blanks created by rinsing and eluting pre-cleaned resins without any prior sample loading. Feature lists for each organism were then aligned to identify both common and unique features among the eight strains tested. Aligned feature lists were also used to investigate intensity level differences among features common to multiple samples.

To provide a rigorous analysis of the similarity between all cultures, an aligned feature list containing all the features detected in every replicate was created and converted into a binary matrix where each column represented an individual culture and each row a feature present in at least one culture. Multiplying this matrix by its transpose created a second matrix where each element was the number of features common to each pairwise culture comparison. The matrix was then normalized by the total number of features present in each pairwise comparison. This normalized similarity matrix was used to quantify the similarity of feature lists and their parent strains, and to generate a cluster diagram as a visual representation of DOM_P_ similarity among strains. The number of features shared by any two cultures was divided by the sum of features present in those cultures to generate a percent similarity value for all pairwise sample combinations.

## Results

We employed liquid chromatography coupled to low-resolution mass spectrometry to characterize DOM produced by eight marine phytoplankton strains grown in axenic batch cultures. We determined this approach to be of sufficient mass resolution based on elemental analyses of DOM using ultrahigh-resolution mass spectrometry that show the following four prominent, reoccurring mass differences: 14.00156 Da (CH_2_), 2.0157 Da (double bond/ring series), 1.0034 Da (^13^C and ^12^C), and 0.0364 Da (C=O vs. CH-CH_3_). All of these mass differences can be distinguished using the mass resolution of the instrument in our study, except for 0.0364 Da. This final mass difference would also likely lead to shifts in retention time that can be resolved by HPLC however, and would therefore be detected as distinct features in our data set. Indeed, we analyzed DOM isolated from *Prochlorococcus* str. MED4 and its corresponding medium control using identical chromatography coupled to Fourier-transform ion cyclotron resonance mass spectrometry (FT-ICR-MS). Processing the resulting data using an appropriate mass resolution (2 ppm) revealed only a modest increase in the number of total DOM features identified in this sample when compared to results obtained using low-resolution mass spectrometry (364 features compared to the 346 features reported in this study; FT-ICR-MS data not shown). Equal sample volumes were injected to minimize differences in signal-to-noise between these analyses.

All eight of the marine phytoplankton strains analyzed produced DOM containing hundreds of extracellular hydrophobic features recovered by SPE and detected by HPLC-ESI-MS (Table 1 and Supplementary File 1). Final cell densities and organic carbon production for each replicate are detailed in Table 2. A variable portion (5–25%) of the organic carbon produced by each phytoplankton strain was released as extracellular DOM where it might be accessible to heterotrophic microbes in a natural setting. The majority of the features recovered were consistently associated with only one of the strains tested, while a relatively small subset appeared to be produced by a diverse range of organisms. Of the 2032 features detected in culture samples, ranging from 104–1466 *m/z*, the majority (1633 features, or 80%) were unique to a particular strain; only a few (6 features, or 0.3%) were found in all eight. Some variation in feature composition was also found among biological replicates for all strains (Table 1)—possibly a result of uncontrollable differences in growth conditions and/or small variations in growth stage at the time of processing. On average, 43% of the features for a given set of triplicates were found in all three, while 24% were found in two out of the three, and the remaining 33% were unique to one replicate. On average, 61% of the features in a given replicate were also present in the other two replicates. DOM_P_ composition of the different strains was compared using two approaches. First, only those features common to all three replicates for a given strain were considered produced by that strain. These features were then compared among all eight strains to stringently identify both common and unique features that were consistently associated with each strain. A second approach employed all features detected in any replicate to generate a similarity matrix for running all possible pairwise comparisons, treating all replicates as individual samples. Both of these approaches identified a similar connection between the phylogenetic relationship of the phytoplankton strains tested and the chemical composition of the DOM they produced.

**Table 2:** **Biomass and organic carbon production by phytoplankton cultures tested in this study**.

**Culture**	**Growth period (days)**	**Maximum cell density (cells/ml)**	**DOC produced (μM)**	**POC produced (mM)**	**DOC produced per cell (fmol)**	**POC produced per cell (fmol)**	**% of total OC released as DOC**	**% DOC recovered via SPE**
MIT9313A	12	6.38E+07	NA	1.0	NA	15.1	NA	NA
MIT9313B	12	6.81E+07	NA	1.0	NA	14.7	NA	NA
MIT9313C	12	6.74E+07	NA	1.0	NA	15.0	NA	NA
MIT9301A	12	2.55E+08	NA	1.1	NA	4.1	NA	NA
MIT9301B	12	3.01E+08	NA	1.2	NA	4.0	NA	NA
MIT9301C	12	2.57E+08	NA	1.1	NA	4.2	NA	NA
MED4B	12	1.63E+08	NA	0.6	NA	3.8	NA	NA
MED4C	12	1.61E+08	NA	0.6	NA	3.7	NA	NA
WH8102A	22	1.06E+08	512.1	1.6	4.8	14.8	25	3
WH8102B	22	1.27E+08	436.7	1.4	3.4	11.2	24	2
WH8102C	22	1.10E+08	499.0	1.6	4.5	14.2	24	3
WH7803A	22	3.14E+07	180.5	0.6	5.7	19.7	23	4
WH7803B	22	4.24E+07	167.8	0.6	4.0	15.1	21	4
WH7803C	22	3.61E+07	172.6	0.7	4.8	18.3	21	6
CCMP1335A	19	1.74E+07	325.3	2.6	18.7	150.6	11	7
CCMP1335B	19	1.06E+07	261.3	2.5	24.7	233.8	10	7
CCMP1335C	19	1.38E+07	239.7	2.5	17.4	182.4	9	9
CCMP1647A	19	1.89E+07	511.5	1.7	27.1	90.9	23	11
CCMP1647B	19	1.95E+07	426.7	1.8	21.9	93.3	19	10
CCMP1647C	19	1.31E+07	358.4	1.5	27.4	117.1	19	11
CCMP632A	19	1.07E+07	172.3	2.8	16.1	264.3	6	22
CCMP632B	19	9.28E+06	147.4	2.8	15.9	302.4	5	24
CCMP632C	19	7.98E+06	188.5	2.8	23.6	356.7	6	24

*Organic carbon values are background subtracted using media controls*.

*“NA” denotes data not available. Organic carbon data for Prochlorococcus strains was not acquired due to the addition of HEPES buffer during growth to mitigate pH changes*.

### Prochlorococcus

**Figure 1:**
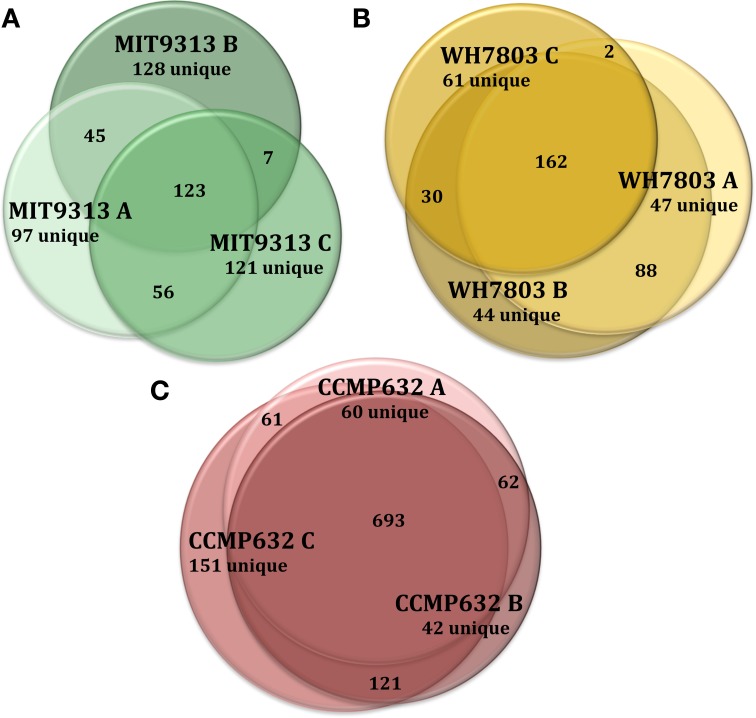
**Venn diagrams showing the degree to which triplicate cultures of representative phytoplankton strains share DOM_P_ features**. Each culture is represented by a circle, the area of which is proportional to the total number of features identified in that sample. The degree of overlap between circles is proportional to the number of shared features. *Prochlorococcus* str. MIT9313 replicates had the most variation of any strain tested **(A)**, while *P. tricornutum* (CCMP632) replicates had the least variation **(C)**. *Synechococcus* str. WH7803 replicates exhibited an average degree of variation indicative of most strains tested in this study **(B)**. For more detailed information regarding replicate correlations, see Table 3.

**Figure 2:**
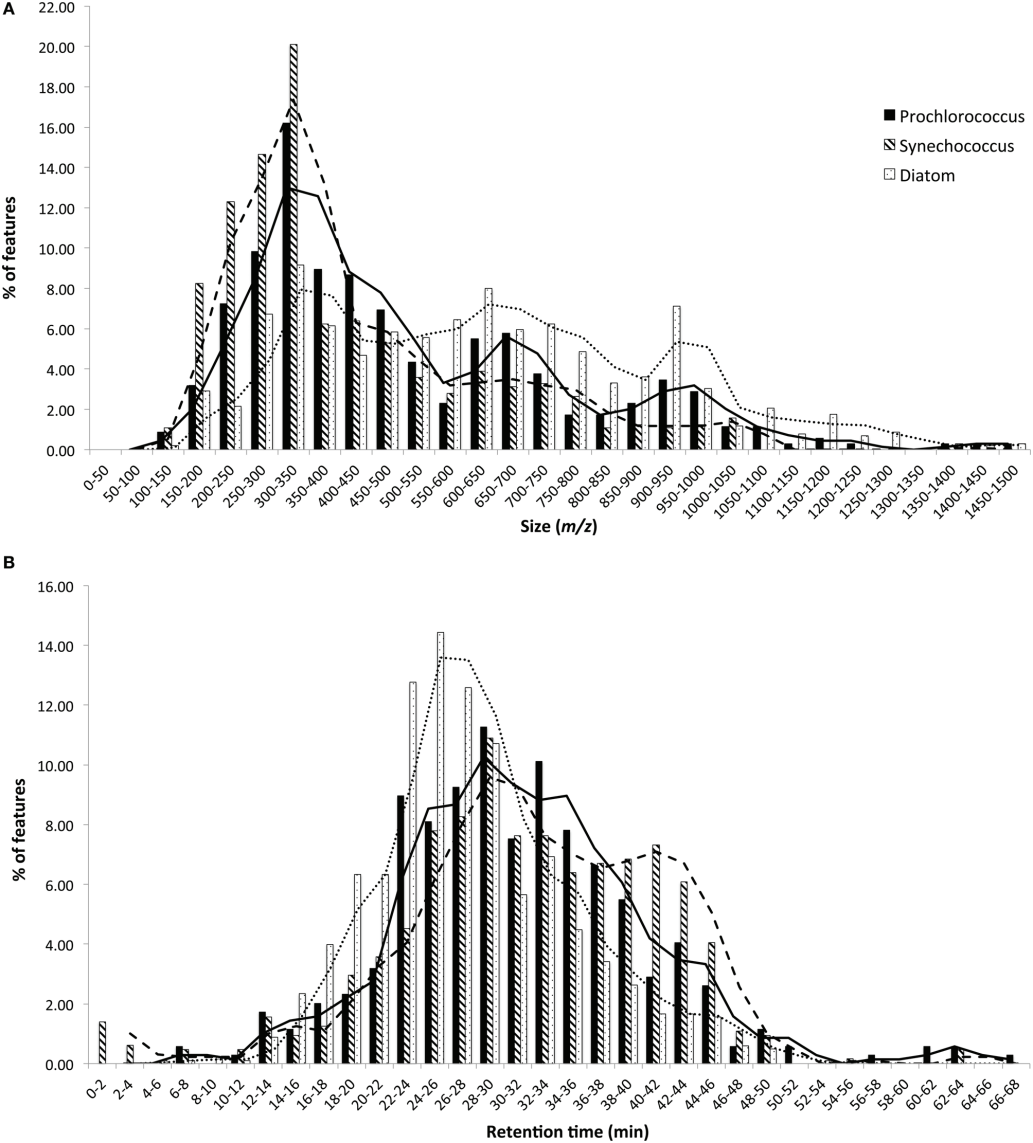
**Histograms comparing the size (A) and polarity (B) distributions of DOM_P_ derived from representative strains from each major phytoplankton group**. Bars indicate the percentage of features that fall within each bin labeled on the X-axes and moving average lines are shown to highlight trends. In general, diatom-derived DOM_P_ had a more even size distribution and a greater abundance of larger, more polar features than cyanobacteria-derived DOM_P_.

HPLC-ESI-MS detected 577, 673, and 463 features in samples from strains MIT9313, MIT9301, and MED4, respectively. Purity broth tests revealed subsequent heterotrophic contamination in one of the MED4 replicate cultures; therefore this sample was removed from all analyses (see N/A in Table 1). Approximately 40% of the features associated with each *Prochlorococcus* strain were detected in all replicates of that strain (Table 1). MIT9313 replicates had the least agreement of all the strains in this study (Figure 1A). *Prochlorococcus*-derived DOM_P_ generally consisted of small, non-polar material, with the majority of features detected between 200–500 *m/z* and 20–50 min, when the mobile phase composition was between 51 and 100% methanol (Figure 2).

**Figure 3:**
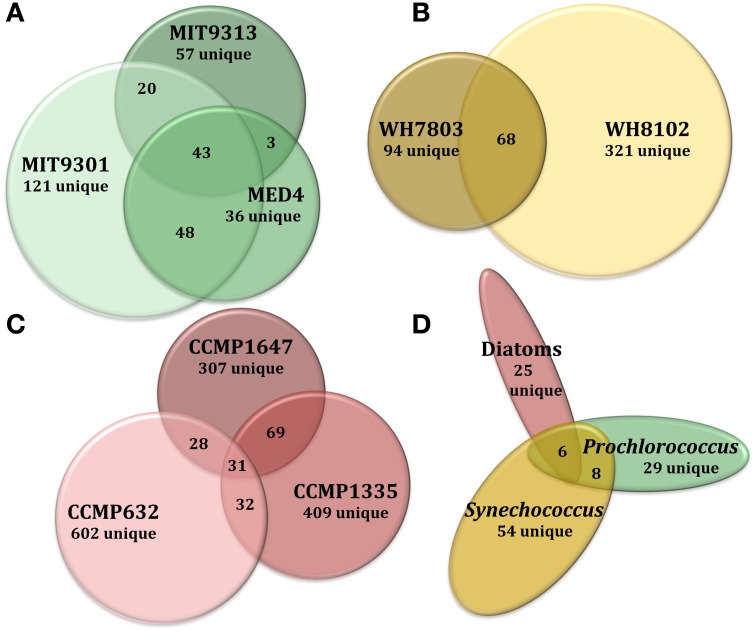
**Venn diagrams comparing DOM_P_ composition at several levels of phylogenetic variation for all eight phytoplankton strains tested in this study**. Features common to all replicate cultures for a given strain are represented by circles or ellipses, the area of which is proportional to the total number of features identified as common for that strain. The degree of overlap between shapes is proportional to the number of shared features between strains of *Prochlorococcus* **(A)**, *Synechococcus* **(B)**, and diatoms **(C)**. Features common to each of these 3 broad phytoplankton groupings were also compared **(D)** to examine variation at the genus and domain levels. For more detailed information regarding percentages of shared DOM_P_ features among different strains, see Table 4.

Of the total *Prochlorococcus*-derived features detected in this study, 13% were produced by all three strains. High-light adapted strains MIT9301 and MED4 were the most similar *Prochlorococcus* strains, sharing 34% of their features. Low-light adapted strain MIT9313 shared approximately 22% of its features with both high-light adapted strains (Figure 3A). Unique features (as much as 52% of the total) were identified in all three strains. Comparing all replicates as individual samples revealed that biological replicates were generally more alike (27–67% similar) than cultures of different strains (12–41% similar), although both MED4 samples (MED4B and MED4C) had greater similarity to replicates of MIT9301 than to each other (Table 3).

**Table 3:** **Percent similarity values for all pairwise comparisons**.

	**MIT9313**	**MIT9313**	**MIT9313**	**MIT9301**	**MIT9301**	**MIT9301**	**MED4**	**MED4**	**WH8102**	**WH8102**	**WH8102**	**WH7803**	**WH7803**	**WH7803**	**CCMP632**	**CCMP632**	**CCMP632**	**CCMP1647**	**CCMP1647**	**CCMPI647**	**CCMP1335**	**CCMP1335**	**CCMPI335**
	**A**	**B**	**C**	**A**	**B**	**C**	**B**	**C**	**A**	**B**	**C**	**A**	**B**	**C**	**A**	**B**	**C**	**A**	**B**	**C**	**A**	**B**	**C**
MIT9313 A	100	37	40	24	20	25	16	25	20	19	20	13	11	8	3	4	3	6	7	6	5	5	4
MIT9313 B		100	27	21	29	24	26	22	15	14	16	10	10	8	3	3	3	5	6	6	4	4	4
MIT9313 C			100	14	14	16	12	19	14	13	13	10	9	7	2	3	3	4	5	4	3	4	4
MIT9301 A				100	38	67	24	33	21	19	22	12	9	8	4	5	5	9	10	9	6	5	5
MIT9301 B					100	48	41	34	17	15	18	10	8	7	3	4	4	7	8	8	5	4	5
MIT9301 C						100	25	36	23	21	23	14	11	8	4	4	5	9	10	10	6	6	6
MED4 B							100	28	11	11	12	7	6	6	3	3	3	5	5	5	3	3	3
MED4 C								100	15	14	17	14	10	8	3	3	3	6	6	6	3	3	3
WH8102 A									100	72	62	19	19	13	5	6	6	11	12	11	8	8	7
WH8102 B										100	58	21	21	14	4	5	5	9	10	9	6	8	7
WH8102 C											100	20	20	14	4	5	5	10	11	12	6	8	8
WH7803 A												100	67	42	4	5	5	5	6	6	5	5	5
WH7803 B													100	50	3	4	4	5	5	6	4	5	4
WH7803 C														100	6	3	4	4	4	5	4	4	4
CCMP632 A															100	73	66	7	8	7	7	7	7
CCMP632 B																100	72	8	9	8	8	9	8
CCMP632 C																	100	8	9	8	9	10	9
CCMP1647 A																		100	61	59	15	15	13
CCMP1647 B																			100	47	14	13	12
CCMP1647 C																				100	13	14	14
CCMP1335 A																					100	71	63
CCMP1335 B																						100	70
CCMP1335 C																							100

*Metabolites present in any replicate media control or blank sample were removed prior to generation of the normalized similarity matrix used to generate these values*.

### Synechococcus

HPLC-ESI-MS detected 773 and 434 features in samples from strains WH8102 and WH7803, respectively. Approximately one half to three quarters of the features associated with each *Synechococcus* strain were detected in all three replicates of that strain (Table 1). The agreement among biological replicates of WH7803 was representative of the average agreement among replicates for all of the strains tested (Figure 1B). The majority of *Synechococcus*-derived features were detected between 150–500 *m/z* and 12–50 min, when the mobile phase composition was between 31 and 100% methanol (Figure 2). Of the total *Synechococcus*-derived features detected in this study, 14% were found associated with both strains. Unique features (as much as 83% of the total) were identified in both strains (Figure 3B). Comparing all replicates as individual samples revealed that all biological replicates were more alike (42–72% similar) than cultures of different strains (13–21% similar) (Table 3).

### Cyanobacteria

Among the features consistently detected in *Prochlorococcus* and *Synechococcus* samples, 14% were found associated with all five cyanobacteria strains. The most similar strains between the two groups were *Prochlorococcus* str. MIT9301 and *Synechococcus* str. WH8102, sharing 20% of their features. All *Prochlorococcus* strains shared more features with each other than with either *Synechococcus* strain. *Synechococcus* str. WH7803 shared more features with the other *Synechococcus* strain tested (WH8102) than with any of the *Prochlorococcus* strains; however the reverse was not true. *Synechococcus* str. WH8102 had the most overlap with *Prochlorococcus* str. MIT9301, sharing 20%, followed by MIT9313 (15%) and then WH7803 (14%). Low-light adapted *Synechococcus* str. WH7803 was more similar to the low-light adapted *Prochlorococcus* str. MIT9313 than it was to either of the high-light adapted *Prochlorococcus* strains. The percent of features shared between all cyanobacteria strain combinations are summarized in Table 4.

**Table 4:**
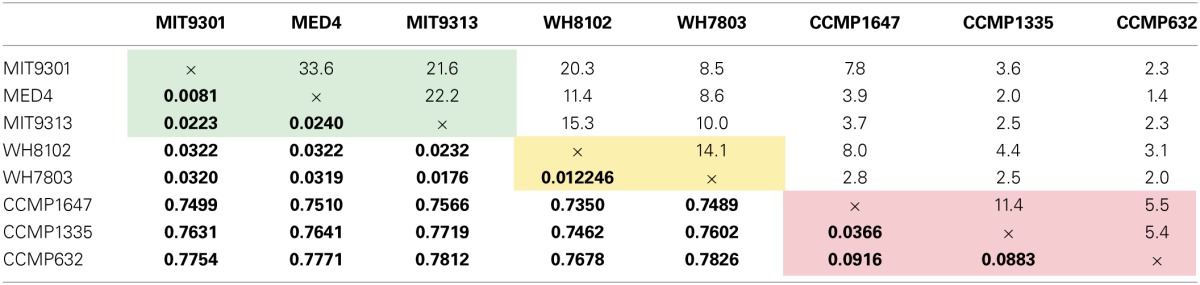
**The percent of features shared among all pairwise strain comparisons (top) alongside sequence distances based on small subunit rRNA gene sequences (bottom in bold) for the eight phytoplankton strains tested in this study**.

*Features used in percentage calculations were considered to be produced by a strain only if they were present in all replicates for that strain and absent in all media controls and blanks. Comparisons between Prochlorococcus strains are highlighted in green, comparisons between Synechococcus strains are highlighted in yellow, and comparisons between diatom strains are highlighted in red*.

*Aligned 16S and 18S rRNA gene sequences were obtained from the SILVA SSURef database release 108, 19.08.2011. The distance matrix was constructed from 1,349 sites using the unweighted LogDet algorithm in Phylip version 3.6.8. See Rocap et al. (2002); Sorhannus (2004) and Kettler et al. (2007) for more information regarding phylogenetic relationships within these groups*.

Comparing all replicates as individual samples confirmed that *Synechococcus* str. WH8102 and *Prochlorococcus* str. MIT9301 were the most similar *Synechococcus* and *Prochlorococcus* strains (15–23% similar) with respect to the DOM features analyzed, and also revealed that WH7803 and the high-light adapted *Prochlorococcus* strains (MIT9301 and MED4) were the least similar cyanobacteria tested (6–14% similar). In general, all of the *Prochlorococcus* strains were more similar to WH8102 (11–23% similar) than WH7803 (6–14% similar) (Table 3).

### Thalassiosira sp.

*T. pseudonana* (CCMP1335) and *T. rotula* (CCMP1647) were chosen to represent different species of the *Thalassiosira* genus. HPLC-ESI-MS detected 973 and 1065 features in samples from *T. pseudonana* and *T. rotula*, respectively. Approximately one half to five sixths of the features associated with each *Thalassiosira* strain were detected in all three replicates of that strain (Table 1). The majority of *Thalassiosira*-derived features were detected between 150–700 *m/z* and 20–45 min, when the mobile phase composition was between 51 and 100% methanol (Figure 2). Of the total *Thalassiosira*-derived features detected in this study, 11% were found associated with both strains. Unique features (as much as 82% of the total) were identified in both strains (Figure 3C). Comparing all replicates as individual samples revealed that all biological replicates were more alike (47–71% similar) than cultures of different strains (12–15% similar) (Table 3).

### Phaeodactylum tricornutum

HPLC-ESI-MS detected 1190 features in *P. tricornutum* samples. Approximately three fourths of the features associated with each *P. tricornutum* culture were detected in all three replicates (Table 1). *P. tricornutum* replicates had the most agreement of all the strains in this study (Figure 1C). *Phaeodactylum*-derived DOM_P_ generally consisted of larger, more polar material when compared to cyanobacteria DOM_P_, with the majority of features detected between 150–1000 *m/z* and 12–45 min, when the mobile phase composition was between 31 and 100% methanol (Figure 2). Comparing all replicates as individual samples revealed that the biological replicates were more alike (66–73% similar) than cultures of any other strain (2–10% similar) (Table 3).

### Diatoms

In general, DOM_P_ composition was more diverse among the diatom strains than among the cyanobacteria. Among the features consistently detected in the diatom strains tested, only 2% were found associated with all three strains. *T. pseudonana* and *T. rotula* were the most similar diatoms, sharing 11% of their features. *T. rotula* and *P. tricornutum* shared 6% of their features, while *T. pseudonana* and *P. tricornutum* shared 5% (Figure 3C). A large number of unique features (as much as 87% of the total) were detected in all three diatom strains. *T. pseudonana* and *T. rotula* were both more similar to each other than to any of the cyanobacteria strains tested and *P. tricornutum* was more similar to both *Thalassiosira* diatoms than to any cyanobacteria strain. Comparing all replicates as individual samples revealed that biological replicates were more alike (47–73% similar) than cultures of different strains (7–15% similar), and confirmed that *T. pseudonana* and *T. rotula* are the most similar diatoms (12–15% similar), followed by *T. pseudonana* and *P. tricornutum* (7–10% similar), and *T. rotula* and *P. tricornutum* (7–9% similar) (Table 3).

### Domain-level comparisons

The 2032 DOM_P_ features detected in this study were organized into three groups: those detected in all *Prochlorococcus* strains, those detected in all *Synechococcus* strains, and those detected in all diatom strains. Of the 122 features that can be classified this way, only 5% (6 features) were common to all three groups. *Prochlorococcus* and *Synechococcus* were the most similar groups, sharing 14% of their features, followed by diatoms and *Prochlorococcus* sharing 10%, and finally diatoms and *Synechococcus* sharing 8% (Figure 3D). All three groups contained a large quantity of features that were unique to that group; the *Synechococcus* group had the most with 54 unique features, or 79% of its total. Combining the *Prochlorococcus* and *Synechococcus* groups into one cyanobacteria group and comparing this with the diatom group reduced the number of total classifiable features to 39. The same 6 features (now 15% of the total) mentioned above were common to both groups, while 8 features (21%) were unique to the cyanobacteria group and 25 features (64%) were unique to the diatom group.

Comparing all replicates as individual samples revealed that all five cyanobacteria strains were more similar to each other (12–41% similar) than to any diatom strain tested (2–12% similar). Comparisons between cyanobacteria and diatom strains revealed *Prochlorococcus* str. MIT9301 and *T. rotula*, and *Synechococcus* str. WH8102 and *T. rotula* to be the most similar strains between these two groups, both sharing 8% of their features. *Prochlorococcus* str. MED4 and *P. tricornutum* were the least similar strains, sharing only 1% of their features. Comparing all replicates as individual samples confirmed that all five cyanobacteria strains were most like *T. rotula* (4–12% similar), followed by *T. pseudonana* (3–8% similar) and finally *P. tricornutum* (2–6% similar). *T. pseudonana* and *P. tricornutum* were both more similar to all diatom strains than to any cyanobacteria tested. While *T. rotula* was most like *T. pseudonana* (11% similar), it had more features in common with *Prochlorococcus* str. MIT9301 (8% similar) and *Synechococcus* str. WH8102 (8% similar) than with *P. tricornutum* (6% similar). All three diatom strains were more like *Synechococcus* str. WH8102 (4–12% similar) than any other cyanobacteria strain (2–10% similar) and more like *Prochlorococcus* str. MIT9301 (3–10% similar) than any other *Prochlorococcus* strain (2–7% similar). Similarity values are summarized in Table 3, while the percent of features shared between strains are shown in Table 4, alongside their rRNA gene sequence distances. Unique features were detected in all eight phytoplankton strains that were not found in any other strain. *P. tricornutum* was the most distinct strain with 587 unique features (49% of its total), while *Prochlorococcus* str. MED4 was the least distinct with 30 unique features (6% of its total). DOM_P_ composition was most similar at the clade-level (14–34% of features in common) and least similar at the domain-level (1–8% of features in common).

Molecular weight and polarity distributions of DOM_P_ were analyzed for additional connections between DOM_P_ composition and phytoplankton phylogeny. While all eight strains produced low-molecular-weight (≤1466 *m/z*) material over a broad polarity spectrum, cyanobacteria-derived DOM_P_ was generally comprised of smaller, less polar features when compared to diatom-derived DOM_P_ (Figure 2). *P. tricornutum* cultures in particular produced large polar DOM, while *T. rotula* produced smaller DOM that more closely resembled cyanobacterial profiles. The low-light adapted *Synechococcus* str. WH7803 produced slightly larger DOM, resembling centric diatom profiles.

### Origin of isolation

While much of the observed variation in DOM_P_ composition appeared related to producer phylogeny, the data also suggested connections between the composition of DOM_P_ and the locations from which these strains were originally isolated (Table 5). Among the diatoms tested, the DOM_P_ composition of *P. tricornutum* was found to be more similar to that of *T. pseudonana*, than to that of *T. rotula*. Both *P. tricornutum* and *T. pseudonana* were isolated from the North Atlantic and under lower temperature conditions than *T. rotula*, which was isolated from the Mediterranean Sea. We also found that DOM_P_ derived from *Prochlorococcus* str. MED4 was more compositionally similar to that of *T. rotula* (the only other strain isolated from the Mediterranean Sea) than to either of the other diatom strains tested. Additionally, DOM_P_ derived from *Prochlorococcus* str. MIT9313 as well both of the *Synechococcus* strains tested was more similar to that of *Prochlorococcus* str. MIT9301 than to that of *Prochlorococcus* str. MED4. MIT9301, MIT9313, and both *Synechococcus* strains were all isolated from the Sargasso Sea and MIT9313 and MIT9301 were both isolated from deeper depths than MED4. *Prochlorococcus* str. MIT9301 and *Synechococcus* str. WH8102 had the highest correlation in DOM_P_ composition between the two cyanobacteria groups. These two strains were originally isolated from the lowest latitudes and most pelagic locations of all the strains tested in this study (Table 5).

**Table 5:** **Isolation and niche information for the eight phytoplankton strains tested in this study**.

**Strain**	**Isolation site**	**Isolation depth (m)**	**Growth temp. (°C)**	**Light optima**
***Prochlorococcus***				
MIT9313	37.5002N 68.2334W Sargasso Sea	135	18–22	Low
MIT9301	34.1667N 66.3000W Sargasso Sea	90	18–22	High
MED4	35.0000N 20.0000E^a^ Mediterranean Sea	5	18–22	High
***Synechococcus***				
WH8102	22.4950N 65.6000W Sargasso Sea	Near surface	22–26	High
WH7803	33.7423N 67.4913W Sargasso Sea	25	22–26	Low
***Diatoms***				
*P. tricornutum*	54.0000N 04.0000W^a^ Eastern N. Atlantic	Near surface	18–22	High
*T. rotula*	40.7500N 14.3300E Mediterranean Sea	Near surface	18–22	High
*T. pseudonana*	40.7560N 72.8200W Western N. Atlantic	Near surface	11–16	High

a *Approximate isolation coordinates*.

*Data compiled from ncma.bigelow.org*.

### Abundance of common features

Although strictly quantitative assessments within a sample are not feasible due to uncertainties in ionization efficiency in ESI, if we assume that matrix effects are minimal and that abundance scales linearly with signal intensity, then the intensity level of features detected in multiple samples offers semi-quantitative information regarding common features associated with different phytoplankton strains. Comparing a particular strain with its respective medium control can identify material present in the seawater-based medium that were also produced by the organism as well as highlight material consumed by phytoplankton during growth.

**Figure 4:**
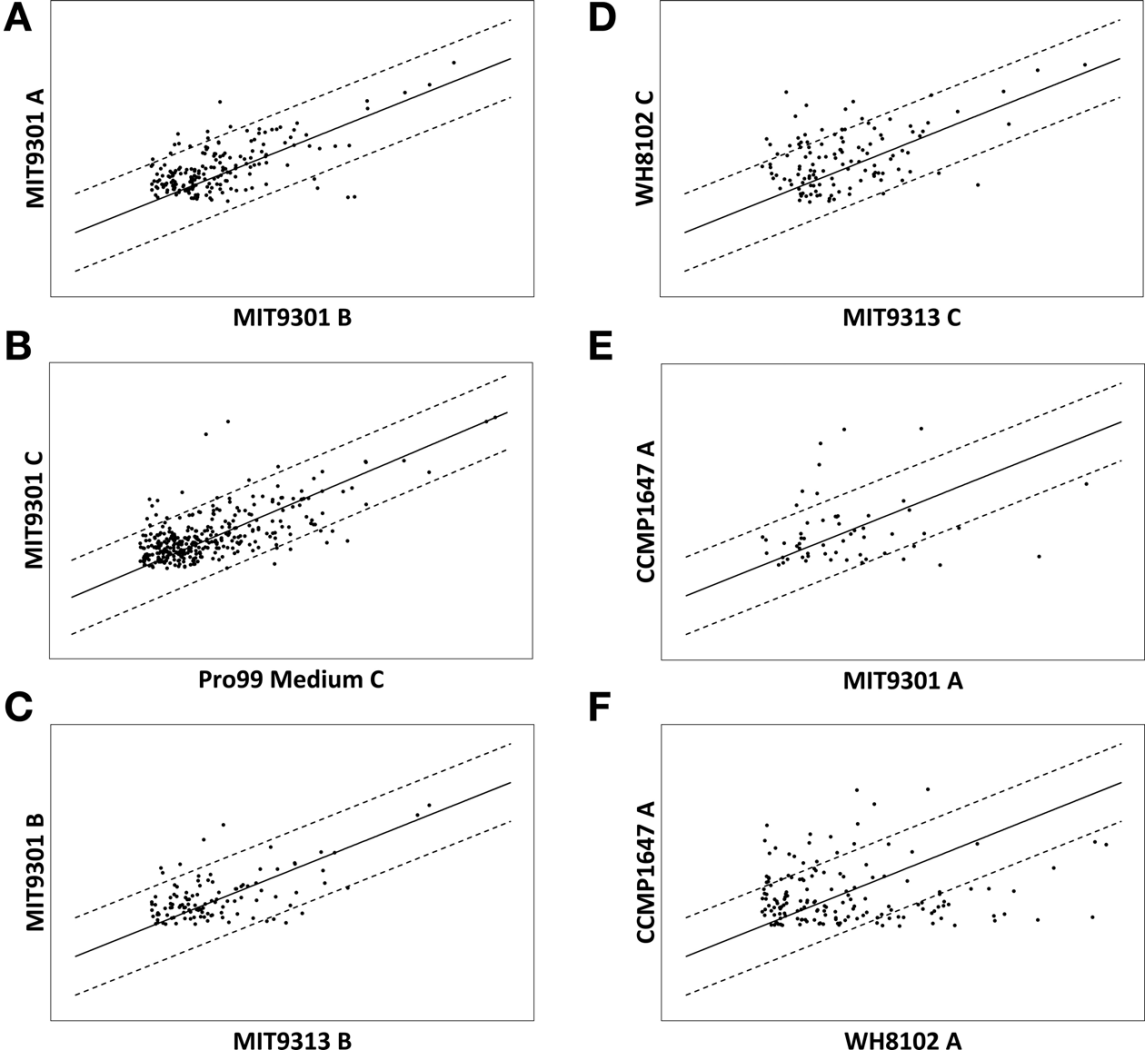
**Scatter plots illustrating variations in signal intensity among features common to multiple samples**. Signal intensity is used as a semi-quantitative proxy for feature abundance. Features are indicated by dots and their location is a function of their signal intensity in the 2 samples labeled on the axes for each plot. Solid lines indicate 1:1 and broken lines indicate 4:1. Variations in common feature intensities are shown for biological replicates **(A)** indicating some abundance variation among replicate samples. Comparing a culture sample to its respective media control **(B)** reveals features present in the medium that are produced (above the upper broken line) and consumed (below the lower broken line) by the phytoplankton strain during growth in culture. Variations in common feature intensities can also be compared at multiple levels of phylogenetic variation including at the clade-level **(C)**, genus-level **(D)**, and domain level **(E,F)**, indicating that the quantity of features produced is also related to producer phylogeny.

Scatter plots of signal intensity provide a visual representation of semi-quantitative differences among features common to multiple samples (Figure 4). Intensity comparisons above and below a threshold of 4-fold were chosen because the majority of common features (93% on average) among all replicate samples and blanks were within a 4-fold intensity difference (Figure 4A). A representative comparison of common features found in a culture sample (*Prochlorococcus* str. 9301 replicate C) and its respective media control (Pro99 medium replicate C) demonstrates that while the majority of features (95% on average) are within a 4-fold intensity difference, there was material present in the medium produced (dots above the upper 4:1 line) and consumed (dots below the lower 4:1 line) during phytoplankton growth (Figure 4B). Pairwise comparisons of different strains exhibited greater intensity differences than among replicates, and the degree of intensity differences between strains varied widely. On average, 91% of common features among different *Prochlorococcus* strains were within a 4-fold intensity difference (Figure 4C). An average of 82% of common features among the different *Synechococcus* strains were within a 4-fold intensity difference, while 77% of common features were within a 4-fold intensity difference among the different diatom strains. On average, 84% of features common to *Prochlorococcus* and *Synechococcus* were within a 4-fold intensity difference, while an average of 75 and 68% of common features were within a 4-fold intensity difference when comparing *Prochlorococcus* to diatoms and *Synechococcus* to diatoms, respectively (Figures 4D–F).

## Discussion

**Figure 5:**
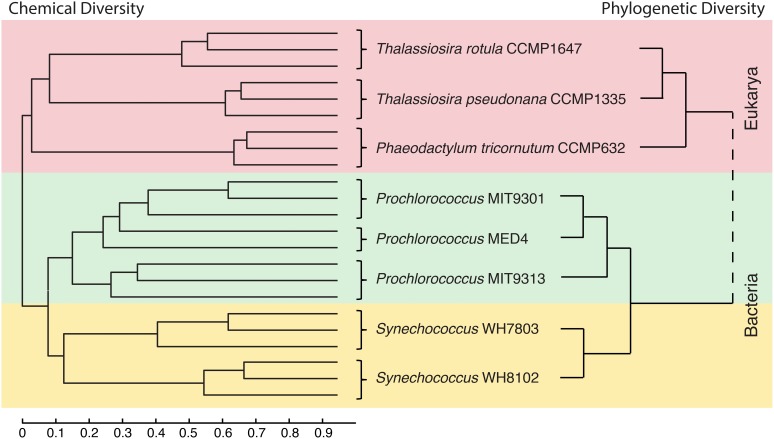
**Phylogeny of the eight model phytoplankton strains and the chemical similarity of the DOM they produce show similar relationship patterns**. At left is shown a dendrogram created from the normalized similarity matrix of all DOM_P_ features identified in each culture replicate after media subtraction using the unweighted pair group method average (UPGMA). The scale bar corresponds to percent similarity values of DOM composition for all pairwise comparisons as given in Table 3. At right is a schematic representation of the phylogenetic relationships between the 8 organisms based on previous work with ribosomal gene sequences and whole genome comparisons (Rocap et al., 2002; Sorhannus, 2004; Kettler et al., 2007). Branch lengths do not correspond to phylogenetic distances. See Table 4, for information regarding rRNA gene sequence distances.

Untargeted metabolomic profiling of DOM_P_ derived from eight model marine phytoplankton strains suggests that producer phylogeny may be an important factor in determining the chemical composition of marine DOM. While a large proportion (80%) of the features detected in this study were unique to a particular strain, cluster analyses based on phylogeny and DOM_P_ composition (by either pooling common features among replicates or analyzing each replicate separately) revealed that phylogenetically related strains tended to produce organic compound suites of a more similar chemical composition (Figure 5). This relationship was found to exist at the domain, order, genus, species, and clade level, and indicates that variations in DOM_P_ composition can reflect phylogenetic relationships among the producing organisms, a potential consequence of connections between their genomes and exometabolomes (defined here as DOM_P_). Chemical trends in the DOM_P_ composition of the phytoplankton strains indicate that eukaryotic phytoplankton may contribute a greater amount of higher molecular weight material over a broad polarity range when compared to cyanobacteria, and that both the molecular weight and polarity of DOM_P_ components may also vary according to biological origin.

The degree of variation in ion intensity among common features was also found to parallel phylogenetic variation. Under the growth conditions tested here, closely related strains not only produced DOM of similar composition, but also produced common components in similar relative abundance (Figure 4). Semi-quantitative analysis in this fashion also provides an opportunity to examine growth dynamics and mixotrophic activity of marine phytoplankton. Many marine phytoplankton have been shown to exhibit mixotrophic tendencies (Cerón García et al., 2006; Bronk et al., 2007; Baran et al., 2011; Gómez-Pereira et al., 2012) and features found in greater abundance in media controls compared to their respective DOM_P_ provide interesting targets for further chemical identification, as these features likely represent compounds present in the growth medium that were consumed by phytoplankton cells during growth (e.g., features below the 4:1 line in Figure 4B).

Our data also lends some support for a possible connection between DOM_P_ composition and habitat. Mass spectrometric analysis revealed similarities in DOM_P_ composition among strains isolated from similar habitats, suggesting that adaptations to a particular environment may also influence DOM production by marine phytoplankton. Although many of the strains employed here have been in laboratory culture for many years, one strain (MED4) has been shown to have an extremely stable genome (Osburne et al., 2010), which we suspect is a characteristic of this phytoplankton group. Thus, these adaptations have likely been retained in current isolates. Genomic and metagenomic studies have shown that environmental conditions can shape the gene content of marine bacteria (Martiny et al., 2009; Coleman and Chisholm, 2010). Potential connections between habitat and DOM_P_ composition appear to be worthy of additional investigation in the field, and many more samples will need to be analyzed to adequately test this hypothesis.

The protocols described here allow for the creation of a spectral database of DOM_P_ characteristics and for identification of target features of interest for additional chemical analyses, such as nuclear magnetic resonance spectroscopy and high-resolution mass spectrometry (Aluwihare et al., 1999; Soule et al., 2010). Spectral data can be compared against existing metabolomic databases for compound identification (Tautenhahn et al., 2012a,b). Each phytoplankton strain that we tested produced and released a complex suite of DOM_P_ containing hundreds to thousands of hydrophobic features. As the effort required to definitively identify a single feature derived from an untargeted metabolomics approach is not trivial (Bowen and Northen, 2010; Schwarz et al., 2013), it is useful to target organic compounds worthy of additional attention. One way to accomplish this task is to organize features into categories based on their presence or absence in particular samples according to phylogenetic grouping. For instance, only six features were found to be common to all of the strains tested here, and likely represent metabolites that are produced by a diverse array of phytoplankton. This relatively short list of features could be likely targets for further chemical characterization. The coupling of DOM_P_ analysis as described here with DOM consumption experiments involving heterotrophic bacterioplankton has the potential to yield novel information regarding chemical transformations of DOM and interactions between autotrophic and heterotrophic marine microorganisms.

Differences in DOM_P_ composition were generally much greater between strains than among biological replicates of the same strain. However, significant variation was found to exist among replicates (Figure 1). While some of these differences may be attributed to variables in sample processing, this finding more likely indicates that DOM_P_ composition is also influenced by factors such as growth conditions, cell density and phase of growth. Growth phase in particular has been shown to affect the composition of DOM released by several diatom strains in culture (Barofsky et al., 2009). Our results support this conclusion, and suggest that even subtle shifts in physiology and other (as yet unidentified) parameters may significantly impact the composition of phytoplankton-derived DOM. To provide sufficient material for analysis, it was necessary to grow the cells to late exponential phase; while all replicates behaved similarly and yielded similar cell densities, slight variations in the final growth phase among replicates may have influenced DOM_p_ composition. The five cyanobacteria strains tested in this study all displayed greater DOM_P_ variation among replicates than the diatom strains, suggesting that cyanobacteria, and *Prochlorococcus* in particular, may have been more susceptible to these factors. Under dynamic *in-situ* conditions, producer phylogeny is likely to be only one of several factors (temperature, light, nutrient availability, growth rate, etc.) influencing the composition of phytoplankton-derived DOM. As the eight strains tested here represent only a small fraction of eukaryotic algal and cyanobacterial diversity grown under controlled conditions, additional studies on an extensive array of marine phytoplankton using continuous cultures or fed batch reactors under a variety of conditions are needed. Further experimentation along these lines will help to determine if marine phytoplankton typically produce DOM suites that reflect their phylogenetic relationships and evaluate how phytoplankton-derived DOM composition varies in response to growth rate and changes in environmental conditions.

Initial results from this study of DOM_P_ show that marine phytoplankton can release characteristic suites of organic material and support the notion that phytoplankton phylogeny is a factor in determining DOM composition. Spatial and temporal variations in phytoplankton community structure (over smaller scales such as seasonal phytoplankton blooms or larger scales including gradual shifts due to global climate conditions) may therefore be accompanied by predictable variations in the composition of marine DOM. Compositional differences in DOM have been shown to affect both the relative abundance and activity of particular heterotrophic bacteria groups, suggesting a relationship between DOM composition and heterotrophic bacterioplankton diversity (Cottrell and Kirchman, 2000; Romera-Castillo et al., 2011; Nelson and Carlson, 2012; Sarmento and Gasol, 2012). If additional studies confirm the relationship between DOM composition and producer phylogeny presented here, then phytoplankton diversity could directly impact heterotrophic bacterioplankton diversity in the marine environment via a DOM link.

### Conflict of interest statement

The authors declare that the research was conducted in the absence of any commercial or financial relationships that could be construed as a potential conflict of interest.
